# Racial Differences in Postpartum Blood Pressure Trajectories Among Women After a Hypertensive Disorder of Pregnancy

**DOI:** 10.1001/jamanetworkopen.2020.30815

**Published:** 2020-12-22

**Authors:** Alisse Hauspurg, Lara Lemon, Camila Cabrera, Amal Javaid, Anna Binstock, Beth Quinn, Jacob Larkin, Andrew R. Watson, Richard H. Beigi, Hyagriv Simhan

**Affiliations:** 1 Magee-Womens Research Institute, University of Pittsburgh School of Medicine, Pittsburgh, Pennsylvania; 2 Department of Obstetrics, Gynecology and Reproductive Sciences, Magee-Womens Hospital, University of Pittsburgh School of Medicine, Pittsburgh, Pennsylvania; 3 Department of Clinical Analytics, University of Pittsburgh Medical Center, Pittsburgh, Pennsylvania; 4 University of Pittsburgh School of Medicine, Pittsburgh, Pennsylvania; 5 Department of Surgery, University of Pittsburgh School of Medicine, Pittsburgh, Pennsylvania

## Abstract

**Importance:**

Maternal morbidity and mortality are increasing in the United States, most of which occur post partum, with significant racial disparities, particularly associated with hypertensive disorders of pregnancy. Blood pressure trajectory after a hypertensive disorder of pregnancy has not been previously described.

**Objectives:**

To describe the blood pressure trajectory in the first 6 weeks post partum after a hypertensive disorder of pregnancy and to evaluate whether blood pressure trajectories differ by self-reported race.

**Design, Setting, and Participants:**

This prospective cohort study included deliveries between January 1, 2018, and December 31, 2019. Women with a clinical diagnosis of a hypertensive disorder of pregnancy were enrolled in a postpartum remote blood pressure monitoring program at the time of delivery and were followed up for 6 weeks. Statistical analysis was performed from April 6 to 17, 2020.

**Main Outcomes and Measures:**

Mixed-effects regression models were used to display blood pressure trajectories in the first 6 weeks post partum.

**Results:**

A total of 1077 women were included (mean [SD] age, 30.2 [5.6] years; 804 of 1017 White [79.1%] and 213 of 1017 Black [20.9%]). Systolic and diastolic blood pressures were found to decrease rapidly in the first 3 weeks post partum, with subsequent stabilization (at 6 days post partum: mean [SD] peak systolic blood pressure, 146 [13] mm Hg; mean [SD] peak diastolic blood pressure, 95 [10] mm Hg; and at 3 weeks post partum: mean [SD] peak systolic blood pressure, 130 [12] mm Hg; mean [SD] peak diastolic blood pressure, 85 [9] mm Hg). A significant difference was seen in blood pressure trajectory by race, with both systolic and diastolic blood pressure decreasing more slowly among Black women compared with White women (mean [SD] peak systolic blood pressure at 1 week post partum: White women, 143 [14] mm Hg vs Black women, 146 [13] mm Hg; *P* = .01; mean [SD] peak diastolic blood pressure at 1 week post partum: White women, 92 [9] mm Hg vs Black women, 94 [9] mm Hg; *P* = .02; and mean [SD] peak systolic blood pressure at 3 weeks post partum: White women, 129 [11] mm Hg vs Black women, 136 [15] mm Hg; *P* < .001; mean [SD] peak diastolic blood pressure at 3 weeks post partum: White women, 84 [8] mm Hg vs Black women, 91 [13] mm Hg; *P* < .001). At the conclusion of the program, 126 of 185 Black women (68.1%) compared with 393 of 764 White women (51.4%) met the criteria for stage 1 or stage 2 hypertension (*P* < .001).

**Conclusions and Relevance:**

This study found that, in the postpartum period, blood pressure decreased rapidly in the first 3 weeks and subsequently stabilized. The study also found that, compared with White women, Black women had a less rapid decrease in blood pressure, resulting in higher blood pressure by the end of a 6-week program. Given the number of women with persistent hypertension at the conclusion of the program, these findings also appear to support the importance of ongoing postpartum care beyond the first 6 weeks after delivery.

## Key Points

**Question:**

Does postpartum blood pressure trajectory after a hypertensive disorder of pregnancy differ by race?

**Findings:**

In this prospective cohort study that included 1077 women after a hypertensive disorder of pregnancy, blood pressure trajectories evaluated using mixed-effects linear regression models differed significantly by self-reported race. At the conclusion of the study, 68% of Black women and 51% of White women met the criteria for stage 1 or stage 2 hypertension.

**Meaning:**

This study suggests that postpartum blood pressure trajectories indicate persistence of higher blood pressures among Black women in this cohort, which may have important implications for postpartum morbidity and mortality associated with hypertensive and cardiovascular conditions in this population.

## Teaser

This cohort study describes the blood pressure trajectory in the first 6 weeks post partum after a hypertensive disorder of pregnancy and evaluates whether blood pressure trajectories differ by self-reported race.

## Introduction

Recent evidence suggests that maternal morbidity and mortality are increasing in the United States, most of which occur in the 6 weeks after delivery, referred to as the “fourth trimester.”^1^ The cause of this increase is likely multifactorial, associated with advancing maternal age and medical comorbidities as well as the concomitant limitations of the health care system. Recent data from the Centers for Disease Control and Prevention^2^ demonstrate that Black women are 3 to 4 times more likely to die in childbirth compared with women of other races/ethnicities, a finding that persists across socioeconomic strata and is thought to be associated with social determinants of health as well as implicit and explicit biases within the health care system, resulting in inequitable treatment.^1,3,4^

Hypertension complicates 10% to 20% of pregnancies in the United States and is significantly associated with maternal morbidity and mortality in the postpartum period.^1,2,5^ Black women are at increased risk of hypertensive disorders of pregnancy, and hypertension and cardiovascular diseases are more frequently associated with morbidity and mortality among Black women compared with White women.^1,3^ These differences are particularly notable in the postpartum period. Despite this finding, prior studies and clinical management guidelines focus overwhelmingly on antepartum and intrapartum management, with relatively little emphasis placed on postpartum management.^6^ One reason for the lack of management guidelines is our limited understanding of the postpartum blood pressure trajectory after delivery and hospital discharge. At present in the United States, women are typically discharged from the hospital on postpartum days 2 to 4 and the American College of Obstetricians and Gynecologists (ACOG) recommends a single blood pressure check between 3 and 10 days post partum for women with a hypertensive disorder of pregnancy.^6,7^ Women with persistent hypertension or the need for titration of antihypertensive medications are typically seen more frequently in the postpartum period for medication management; however, as there are no clear guidelines on optimal blood pressure management in this period, this varies by clinician and institution.^8^ Subsequently, women are typically seen at 4 to 6 weeks post partum for a comprehensive postpartum visit and referred to their primary care physician if there are additional needs for antihypertensive medication management.^6^ This strategy is limited by poor adherence to follow-up, with prior studies showing visit attendance rates of 45% to 60% in this period.^7,9^

The ACOG and others have suggested the use of telemedicine or remote health care interventions to facilitate care in the fourth trimester.^7^ A previous study has demonstrated feasibility, high engagement, retention, and patient satisfaction with a postpartum hypertension remote monitoring program as well as improved adherence with postpartum visits.^10^ Given poor adherence to recommended follow-up in the postpartum period and resultant lack of longitudinally collected serial blood pressure measurements, the trajectory of a decrease in blood pressure in the postpartum period has not been previously well studied, to our knowledge. Prior work has focused on small historic cohorts or a predominantly White population.^11,12,13^ Therefore, the aims of this prospective cohort study were 2-fold. The first aim was to describe the trajectory of postpartum blood pressure after a hypertensive disorder of pregnancy using remote blood pressure monitoring in a large, contemporaneous cohort. The second aim, given the disparities in maternal morbidity and mortality associated with hypertensive disorders in the postpartum period, was to investigate differences in postpartum blood pressure trajectory by race.

## Methods

This is an ongoing quality improvement project that included women admitted to the postpartum unit of a single tertiary care hospital (University of Pittsburgh Medical Center [UPMC] Magee-Womens Hospital) between January 1, 2018, and December 31, 2019. Eligible women had 1 of the following hypertension-related diagnoses: gestational hypertension, preeclampsia, eclampsia, or new-onset postpartum hypertension. Women with prepregnancy chronic hypertension were excluded. To be included in the program, women must have been English-speaking and have access to a text messaging–enabled mobile device. Diagnoses were made by the clinical care team according to ACOG criteria.^6^ Regardless of diagnosis, hypertension in pregnancy was defined as blood pressure of 140 mm Hg or more systolic or 90 mm Hg or more diastolic. Maternal, obstetric, and sociodemographic data were obtained from the electronic medical record and subsequently the Clinical Data Warehouse at UPMC. The program was approved by the UPMC Quality Improvement Review Committee and the University of Pittsburgh institutional review board, which waived a requirement for informed consent because the data were deidentified. The reporting followed the Strengthening the Reporting of Observational Studies in Epidemiology (STROBE) reporting guideline for cohort studies.

A description of the remote monitoring program has been previously described.^10^ In brief, we created a remote patient monitoring platform that used Vivify Health as its core vendor. The monitoring platform was integrated with electronic health records and the Clinical Data Warehouse for both ordering and results. Patients were enrolled in the program by their primary obstetric clinician while admitted as inpatients on the postpartum unit or after a postpartum readmission. After identification and verification of eligibility, the clinician placed an order in the electronic medical record with the patient’s telephone number, which automatically generated a text message to enroll the patient. We used the A&D UA-651 (A&D Medical) automatic upper arm blood pressure monitor. The patient was trained on the use of the blood pressure device by a program-specific nurse educator prior to discharge from the hospital. During program enrollment, bedside nursing staff recorded blood pressure on both the home blood pressure monitoring device and the hospital device to confirm accuracy. The program was managed through a nursing-staffed UPMC call center with documentation of telephone calls and blood pressures directly into the electronic medical record.

We designed nursing call center–driven blood pressure management and treatment algorithms that were developed by local expert stakeholders, consistent with national guidelines on goals for postpartum hypertension management.^14^ After discharge from the hospital, women were prompted to check their blood pressure 5 days per week for the first week of the program and between 3 and 5 times per week for the remainder of the program through 6 weeks post partum. Blood pressures were reported using text messaging. The initial choice of antihypertensive agent was dictated by the clinical care team while the patient was an inpatient. After hospital discharge, titration of medication or, in the case of medication initiation, selection of the antihypertensive agent was based on clinical judgment from the call center physician. There are currently no standardized management guidelines for specific antihypertensive agents or parameters for medication titration in the postpartum period.^8^ Women with symptoms including chest pain, severe headache, blurry vision or vision disturbances, shortness of breath, or blood pressures of 180 mm Hg or more systolic or 110 mm Hg or more diastolic were referred to the emergency department for further evaluation. Discontinuation or downward titration of antihypertensive therapy is based on patients having 3 consecutive blood pressures less than 120/70 mm Hg.

### Statistical Analysis

For the first objective, mixed-effects regression models were used to display blood pressure trajectories in the first 6 weeks post partum, with weeks post partum as the timescale for all analyses. We used repeated blood pressure measurements to fit mixed-effects linear regression models with each blood pressure measurement as the outcome, participant identifier as random intercepts, and weeks post partum as a fixed effect expressed using restricted cubic splines with 4 knots positioned at 0.6, 1.9, 3.6, and 5.9 weeks. The optimal number of knots was chosen comparing both Akaike information criterion and bayesian information criterion between models. Mixed-effects regression models were further adjusted for predefined covariates known to be associated with blood pressure, including prepregnancy body mass index (BMI; calculated as weight in kilograms divided by height in meters squared), tobacco use, type of hypertensive disorder, and use of antihypertensive medication.

For the second objective, we repeated all models stratified by race (White individuals vs Black individuals). Stratified mixed-effects regression models were again adjusted for the same covariates. The number and position of splines were not different by race. In addition, in sensitivity analyses we repeated all analyses including gestational weight gain and BMI at the time of delivery. Differences between races in the associations between blood pressure and weeks post partum were tested via likelihood ratio test between models with and without parameters representing the interaction between race and prepregnancy BMI, BMI at delivery, and gestational weight gain based on results of prior studies.^13,15^ In sensitivity analyses, we repeated all regressions stratified by type of hypertensive disorder, use of antihypertensive medication, and obesity, as each could modify the association.

Statistical analysis was performed from April 6 to 17, 2020. All analyses were performed using Stata IC, version 16 software package (StataCorp LP). All *P* values were from 2-sided tests, and results were deemed statistically significant at *P* < .05.

## Results

A total of 1114 women (mean [SD] age, 30.1 [5.6] years; and mean [SD] BMI, 29.6 [7.9]) were enrolled in the program between January 1, 2018, and December 31, 2019; 37 women reported less than 2 blood pressure measurements and were excluded, leaving 1077 in the analytic sample. For the first objective, we included all 1077 women, who contributed 17 146 blood pressure measurements across the first 6 weeks post partum. Overall, women contributed a mean (SD) of 20.3 (7.1) blood pressure values during the program. The demographic and delivery characteristics of the overall cohort are shown in eTable 1 and eTable 2 in the Supplement. Overall, in our cohort, 447 women (41.5%) had gestational hypertension, and 630 women (58.5%) had preeclampsia, and of these 1077 women, 315 (29.2%) had preeclampsia with severe features.

**Figure 1.**
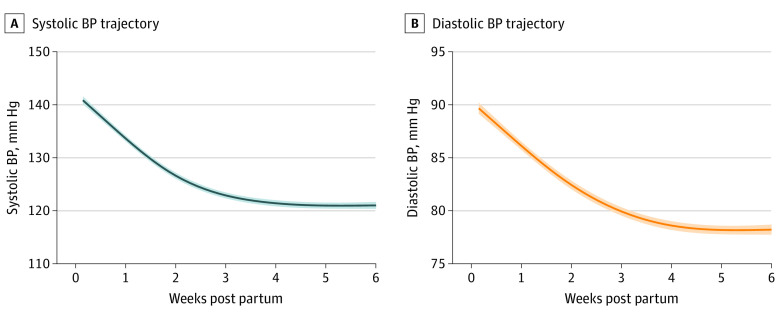
Fitted Blood Pressure (BP) Trajectory in First 6 Weeks Post Partum Shaded area indicates 95% CI.

Figure 1 displays fitted blood pressure values for all participants during the first 6 weeks post partum. The mean (SD) peak systolic blood pressure for the cohort was 146 (13) mm Hg at a median of 6 days post partum (interquartile range [IQR], 4-9 days). Mean (SD) peak diastolic blood pressure at a median of 6 days post partum (IQR, 4-11 days) was of 95 (10) mm Hg. We observed a rapid decrease in blood pressure in the first 3 weeks post partum (mean [SD] peak systolic blood pressure, 130 [12] mm Hg; and mean [SD] peak diastolic blood pressure, 85 [9] mm Hg). Subsequently, blood pressures stabilized for the remaining 3 weeks of the program. Overall among women with a postpartum visit blood pressure value available or who reported a blood pressure value after the third week post partum (n = 1007 [93.5%]), 543 women (53.9%) met criteria for stage 1 hypertension and 200 women (19.9%) met criteria for stage 2 hypertension at the conclusion of the program.

**Figure 2.**
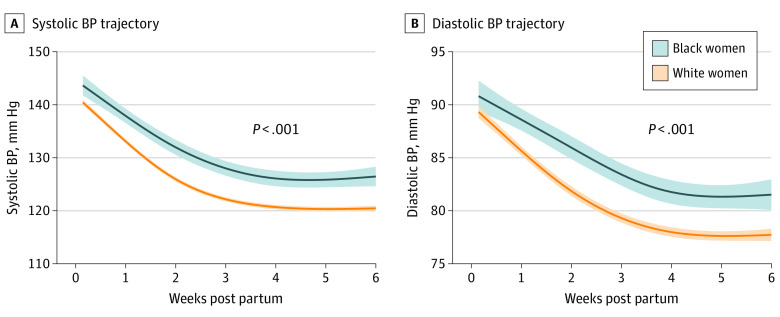
Fitted Blood Pressure (BP) Trajectory in First 6 Weeks Post Partum by Race Shaded area indicates 95% CI.

For our second objective, we excluded 60 women of non-White or non-Black race. Compared with White women (n = 804), Black women (n = 213) were younger (mean [SD] age, 28.1 [6.1] vs 30.6 [5.4] years) and were more likely to have public health insurance (139 [65.3%] vs 209 [26.0%]), be multiparous (107 [50.2%] vs 303 [37.7%]), and to have lower mean (SD) diastolic blood pressure at the initiation of prenatal care (70 [9] vs 74 [8] mm Hg) (Table 1). Infants born to Black women had a lower mean (SD) birth weight than those born to White women (2849 [800] vs 2966 [743] g) (Table 2). There were no differences by race in gestational age at delivery. There were also no differences by race in the highest blood pressure in the 24 hours prior to hospital discharge. Black women had higher peak systolic and diastolic blood pressures compared with White women (mean [SD] maximum systolic blood pressure, 150 [14] vs 145 [13] mm Hg; *P* < .001; and mean [SD] maximum diastolic blood pressure, 98 [12] vs 94 [9] mm Hg; *P* < .001). In addition, Black women reached peak blood pressures at slightly later time points post partum compared with White women (median time of maximum systolic blood pressure, 8 days [IQR, 5-15 days] vs 7 days [IQR, 4-10 days]; and median time of maximum diastolic blood pressure, 9 days [IQR, 5-18 days] vs 7 days [IQR, 5-12 days]). As shown in Figure 2, both systolic and diastolic blood pressure decreased more slowly in the first 6 weeks post partum among Black women compared with White women (mean [SD] peak systolic blood pressure at 1 week post partum: White women, 143 [14] mm Hg vs Black women, 146 [13] mm Hg; *P* = .01; mean [SD] peak diastolic blood pressure at 1 week post partum: White women, 92 [9] mm Hg vs Black women, 94 [9] mm Hg; *P* = .02; and mean [SD] peak systolic blood pressure at 3 weeks post partum: White women, 129 [11] mm Hg vs Black women, 136 [15] mm Hg; *P* < .001; mean [SD] peak diastolic blood pressure at 3 weeks post partum: White women, 84 [8] mm Hg vs Black women, 91 [13] mm Hg; *P* < .001). In analyses adjusting for clinical covariates (including prepregnancy BMI, tobacco use, type of hypertensive disorder, and use of antihypertensive medication), our findings were unchanged. We tested for interaction between race and prepregnancy BMI and found that the interaction term was not statistically significant. In addition, we tested for interaction between race and type of hypertensive disorder, preterm hypertensive disorder, and parity and found no evidence of an interaction. Multivariable-adjusted blood pressure trajectories remained higher in Black women compared with White women during the first 6 weeks post partum (likelihood ratio test χ^2^ = 36.9 for systolic blood pressure; and χ^2^ = 42.8 for diastolic blood pressure; *P* < .001 for all). At the conclusion of the program, mean blood pressure was higher among Black women compared with White women (mean [SD] systolic blood pressure, 131 [14] vs 122 [11] mm Hg; *P* < .001; and mean [SD] diastolic blood pressure, 84 [12] vs 79 [9] mm Hg; *P* < .001). Similarly, Black women were significantly more likely than White women to meet criteria for stage 1 or stage 2 hypertension at the conclusion of the program (126 of 185 [68.1%] vs 393 of 764 [51.4%]; *P* < .001) (Table 3).

**Table 1.** Demographic Characteristics by Self-reported Race

Characteristic	White (n = 804)	Black (n = 213)	*P* value
Age, mean (SD), y	30.6 (5.4)	28.1 (6.1)	<.001
Prepregnancy BMI, mean (SD)	29.4 (7.8)	30.6 (8.5)	.07
Delivery BMI, mean (SD)	34.5 (6.8)	35.4 (7.5)	<.001
Gestational weight gain, mean (SD), kg	14.2 (10.4)	12.2 (12.1)	<.001
Gestational age at prenatal care establishment, mean (SD), wk	11.1 (6.3)	12.5 (6.6)	.007
First prenatal systolic BP, mean (SD), mm Hg	118 (11)	119 (10)	.12
First prenatal diastolic BP, mean (SD), mm Hg	74 (8)	70 (9)	<.001
Insurance status, No. (%)			
Private insurance	579 (72.0)	71 (33.3)	<.001
Public insurance	209 (26.0)	139 (65.3)	
Other	16 (2.0)	3 (1.4)	
Primiparous, No. (%)	501 (62.3)	106 (49.8)	<.001
Current tobacco use, No. (%)	81 (10.1)	33 (15.5)	.02
Pregestational diabetes, No. (%)	24 (3.0)	11 (5.2)	.12
Gestational diabetes, No. (%)	82 (10.2)	22 (10.3)	.35

Abbreviations: BMI, body mass index (calculated as weight in kilograms divided by height in meters squared); BP, blood pressure.

**Table 2.** Delivery and Postpartum Characteristics by Self-reported Race

Characteristic	White (n = 804)	Black (n = 213)	*P* value
Type of hypertension, No. (%)			
Gestational hypertension	350 (43.5)	81 (38.0)	.15
Preeclampsia	454 (56.5)	132 (62.0)	
Cesarean delivery, No. (%)	343 (42.7)	101 (47.4)	.21
Birth weight, mean (SD), g	2966 (743)	2849 (800)	.05
Gestational age at delivery, mean (SD), wk	37.3 (2.7)	37.4 (3.2)	.71
Highest systolic BP in 24 h prior to hospital discharge, mean (SD), mm Hg	140 (12.6)	140 (11.6)	.47
Highest diastolic BP in 24 h prior to hospital discharge, mean (SD), mm Hg	88 (7.1)	88 (7.4)	.59
Discharged with prescription for antihypertensives, No. (%)^a^	183 (22.8)	63 (25.6)	
β-Blocker	77 (9.6)	23 (10.8)	.04
Calcium channel blocker	119 (14.8)	42 (19.7)	
Other	2 (0.2)	0	
Maximum systolic BP, mean (SD), mm Hg	145 (13)	150 (14)	<.001
Maximum diastolic BP, mean (SD), mm Hg	94 (9)	98 (12)	<.001
Time of maximum systolic BP, median (IQR), days post partum	7 (4-10)	8 (5-15)	.02
Time of maximum diastolic BP, median (IQR), days post partum	7 (5-12)	9 (5-18)	.002
Hospital readmission, No. (%)	76 (9.5)	36 (16.9)	.02
Seen for postpartum appointment, No. (%)	686 (87.8)	144 (72.0)	<.001
Number of blood pressures reported through program, median (IQR)	17 (11-22)	14 (7-19)	<.001
Last postpartum week reported, median (IQR)	6 (5-6)	6 (3-6)	<.001
BP recorded after week 3 postp artum, No. (%)	764 (95.0)	185 (86.9)	<.001

Abbreviations: BP, blood pressure; IQR, interquartile range.

^a^ Women may be discharged with more than 1 antihypertensive agent.

**Table 3.** Mean Blood Pressure and Blood Pressure Category by Race at Conclusion of Program Among Women With a Blood Pressure Measured After 3 Weeks Post Partum

Characteristic	White (n = 764)	Black (n = 185)	*P* value
Systolic blood pressure, after week 3, mean (SD), mm Hg	120 (11)	127 (14)	<.001
Diastolic blood pressure, after week 3, mean (SD), mm Hg	78 (9)	81 (11)	<.001
Stage 1 hypertension (≥130-139/80-89 mm Hg), No. (%)	393 (51.4)	126 (68.1)	<.001
Stage 2 hypertension (≥140/90 mm Hg), No. (%)	139 (18.2)	60 (32.4)	<.001

Sensitivity analyses were stratified by antihypertensive medication use, type of hypertensive disorder, and obesity. We observed similar findings in both the unadjusted and adjusted models (eFigures 1-4 in the Supplement).

Finally, to understand race-specific blood pressure trajectories in context, we examined the incidence of hospital readmission for hypertension in the postpartum period and observed the incidence to be higher among Black women compared with White women during the first 6 weeks post partum (36 of 213 [16.9%] vs 76 of 804 [9.5%]; *P* = .02). There were no maternal deaths in the cohort during this period.

## Discussion

In this study, we found that, in the postpartum period, blood pressure decreased rapidly in the first 3 weeks and subsequently stabilized, although a substantial proportion of women continued to have hypertension at 6 weeks post partum. We also found that, compared with White women, Black women had a less rapid decrease in blood pressure post partum, resulting in higher blood pressure by the end of the 6-week program. By 6 weeks post partum, 68.1% of Black women met criteria for stage 1 or stage 2 hypertension, compared with 51.4% of White women (*P* < .001). Our findings have potential implications for addressing postpartum maternal morbidity and mortality, as well as longer-term cardiovascular health, in this population. In this study, there were no differences by race in early pregnancy blood pressure or in the highest blood pressure prior to hospital discharge. However, the more adverse postpartum blood pressure trajectories seen in Black women are consistent across various sensitivity analyses and translate to a higher incidence of hypertension-related hospital readmission.

There are several possible explanations for our findings. In previous studies evaluating the association of race with cardiovascular recovery after peripartum cardiomyopathy, cardiac recovery takes twice as long in Black women compared with White women.^16,17^ Recent studies have demonstrated the causal overlap between peripartum cardiomyopathy and hypertensive disorders of pregnancy.^18,19^ It is well documented both within our data as well as in prior studies that hypertension is exacerbated at 3 to 7 days post partum.^20^ The cause of this exacerbation is unclear; however, others have speculated that it may be due to mobilization of fluid during this period.^20^ If these fluid shifts result in volume overload or subclinical heart failure, perhaps our findings may mirror the differences seen by race in peripartum cardiomyopathy. This finding would suggest that additional postpartum diuresis might be particularly important in this population, as has been demonstrated by recent work in a predominantly African American population.^21^ Our findings are consistent with recent work from Lopes Perdigao et al,^13^ who demonstrated in a cohort of 84 women that women with higher BMI and of Black race had higher mean blood pressure values and were less likely to have resolution of their hypertension by 16 days post partum.

Within the context of small randomized clinical trials or research studies, text message–based postpartum hypertension monitoring programs have been shown to increase patient engagement and improve adherence with blood pressure follow-up.^9,22^ A previous study has demonstrated the feasibility of, high engagement with, retention in, and patient satisfaction with our remote monitoring program as well as improved adherence with postpartum visits.^10^ Given poor adherence to recommended follow-up in the postpartum period, the trajectory of a decrease in blood pressure post partum has not been well studied previously. This trajectory may be particularly important in consideration of rising maternal morbidity and mortality in the postpartum period.^1^ Furthermore, prior studies have shown lower adherence to recommended follow-up among Black women and women of lower socioeconomic status.^7,23^

The American Heart Association and ACOG have identified hypertensive disorders of pregnancy as risk factors for later-life cardiovascular disease; however, effective evidence-based interventions have not yet been adequately studied or implemented, to our knowledge.^24,25,26,27^ Prior studies have shown that postpartum care after a hypertensive disorder of pregnancy is often fragmented, without a systematic transition from the obstetrician to an internist or cardiologist.^25^ Given the fragmented care and poor attendance rates, our findings raise the question of whether universal home blood pressure monitoring and management with antihypertensive therapy for women with persistent hypertension beyond the first 6 weeks post partum may be warranted. This is particularly important given the proportion of women in our cohort with ongoing hypertension at 6 weeks post partum and our finding that Black women with a hypertensive disorder ended the program with systolic blood pressures 9 mm Hg higher systolic and diastolic blood pressures 5 mm Hg higher than White women. More important, there were no differences by race in prenatal blood pressure and our findings suggest that a hypertensive disorder of pregnancy may have an adverse association with blood pressure. We cannot assess from our findings whether this persistent postpartum hypertension represents new-onset chronic hypertension or the ongoing resolution of the hypertensive disorder of pregnancy. For women with public health insurance, in many states within the United States, Medicaid coverage lasts only through 60 days post partum. Most maternal deaths occur in the postpartum period, with approximately one-third occurring after 6 weeks post partum.^2^

### Strengths and Limitations

Our study has several strengths. This was a large, prospective cohort from a single tertiary care center. Participants provided real-time blood pressure measurements using validated, calibrated blood pressure monitors. We used standardized management protocols to reduce the impact of clinician variability in management approaches. Our inclusion of prenatal and in-hospital postpartum blood pressure values also strengthens our findings.

Our study also has several limitations. Participants were enrolled in the program by a clinical care professional. We cannot exclude the possibility that women enrolled in the program differ from those who were not enrolled in the program or that implicit bias or structural racism were associated with participant enrollment in the program. The program requires that women have access to a text messaging–enabled telephone. Prior studies have shown that approximately 92% to 96% of reproductive-aged women have access to a smartphone.^28^ However, it is possible that a larger proportion of high-risk women may not have access to a text- messaging–enabled phone; thus, the applicability of our findings to a broader population within the United States may be limited. The single-site nature of the study limits the generalizability of our findings. In addition, all blood pressure values are self-reported through the program; thus, we cannot exclude the possibility that participants may not report accurate measurements, as no external validation was performed. As with all analytic approaches, mixed-effects regression modeling has several limitations. However, owing to the structure of our data and the ability to account for some heterogeneity over time, this was the most appropriate approach. Black women reported statistically fewer blood pressure values through the program and reported their final blood pressure value at an earlier postpartum time than White women. Although these differences are statistically significant, the clinical significance is likely less important given the overall number of blood pressure values reported and the median follow-up time across both groups with more than 85% follow-up beyond 3 weeks. However, we cannot exclude the possibility that our findings may be associated with bias in attendance vs nonattendance at the postpartum visit and adherence to the remote monitoring program.

Our remote monitoring program concludes at 6 weeks post partum; thus, a further decrease in blood pressure beyond this period cannot be assessed. Furthermore, our cohort includes only women with a hypertensive disorder of pregnancy. We cannot assess how these blood pressure trajectories may differ from those of women without a diagnosed hypertensive disorder. Outside of pregnancy, multiple randomized clinical trials have shown differing effects of antihypertensive agents by race, such that the American College of Cardiology and American Heart Association guidelines recommend calcium channel blockers as first-line antihypertensive agents in African Americans.^29,30^ These recommendations have not been assessed in a pregnant or postpartum population. Within our cohort, β-blockers and calcium channel blockers were used equally among Black and White patients. Although our findings persisted among women not taking any antihypertensive agent, we cannot exclude the possibility that the interaction of race and type of antihypertensive agent used could have been associated with our results. Further study is needed to assess whether findings regarding race and effectiveness of specific antihypertensive agents are upheld in a pregnant and postpartum population. Finally, race is a heterogenous and multifaceted exposure that in our health care system encompasses an economic, social, and physiological construct that includes both biological and nonbiological aspects. It is possible that our findings are secondary to other unmeasurable factors such as implicit and explicit bias within the structure of our medical institutions or the remote monitoring program itself. Unfortunately, because we used data from a program built into clinical care, we are unable to assess whether there are management differences in response to elevated blood pressures by race. The structure of the program does not allow for accurate ascertainment of antihypertensive medication changes, as they are frequently changed through a verbal recommendation rather than through issuing a new prescription or documentation in the medical record. Blood pressure management through the program follows a standard protocol as described previously and information regarding patient race was not available to the on-call physicians managing blood pressure through the program.^10^ Thus, we are unable to assess whether variable treatment based on race could have been associated with our findings, but this remains of particular concern given prior work suggesting differential treatment based on race^31,32,33^ and remains an area of active investigation for our team. We also continue to investigate whether the differences we see in blood pressure trajectory are associated with the racial disparities in morbidity and mortality associated with hypertensive and cardiovascular conditions.

## Conclusions

The findings of this cohort study suggest that, compared with White women, Black women have a postpartum blood pressure trajectory with persistence of higher blood pressure values that may be associated with the increased risk of morbidity and mortality seen in this period. Given the difficulty with adherence to care in this period, our findings support the use of remote monitoring among women at highest risk for hypertensive-related morbidity and suggest the importance of ongoing access to care beyond 6 weeks post partum.

## Notes

Supplement. **eTable 1.** Demographics of Overall Cohort **eTable 2.** Delivery and Postpartum Characteristics **eFigure 1.** Fitted BP Trajectory (Solid Line) and 95% CI (Shaded Area) in First 6 Weeks Postpartum Among Obese Women (BMI ≥30 kg/m^2^) by Race **eFigure 2.** Fitted BP Trajectory (Solid Line) and 95% CI (Shaded Area) in First 6 Weeks Postpartum Among Women on No Antihypertensive Medications by Race **eFigure 3.** Fitted BP Trajectory (Solid Line) and 95% CI (Shaded Area) in First 6 Weeks Postpartum Among Women on Antihypertensive Medications at the Time of Discharge by Race **eFigure 4.** Fitted BP Trajectory (Solid Line) and 95% CI (Shaded Area) in First 6 Weeks Postpartum Among Women With Preeclampsia by Race
