# Supplementary material for: Racial Differences in Postpartum Blood Pressure Trajectories Among Women After a Hypertensive Disorder of Pregnancy
Source: JAMA Netw Open. 2020 Dec 22;3(12):e2030815. doi: 10.1001/jamanetworkopen.2020.30815 (PMC7756239; doi:10.1001/jamanetworkopen.2020.30815)
Supplement: Supplement. — eTable 1. Demographics of Overall Cohort eTable 2. Delivery and Postpartum Characteristics eFigure 1. Fitted BP Trajectory (Solid Line) and 95% CI (Shaded Area) in First 6 Weeks Postpartum Among Obese Women (BMI ≥30 kg/m2) by Race eFigure 2. Fitted BP Trajectory (Solid Line) and 95% CI (Shaded Area) in First 6 Weeks Postpartum Among Women on No Antihypertensive Medications by Race eFigure 3. Fitted BP Trajectory (Solid Line) and 95% CI (Shaded Area) in First 6 Weeks Postpartum Among Women on Antihypertensive Medications at the Time of Discharge by Race eFigure 4. Fitted BP Trajectory (Solid Line) and 95% CI (Shaded Area) in First 6 Weeks Postpartum Among Women With Preeclampsia by Race [file jamanetwopen-e2030815-s001.pdf]

Supplementary Online Content

Hauspurg A, Lemon L, Cabrera C, et al. Racial differences in postpartum blood pressure trajectories among women after a hypertensive disorder of pregnancy. *JAMA Netw Open.* 2020;3(12):e2030815. doi:10.1001/jamanetworkopen.2020.30815

eTable 1. Demographics of Overall Cohort
eTable 2. Delivery and Postpartum Characteristics
eFigure 1. Fitted BP Trajectory (Solid Line) and 95% CI (Shaded Area) in First 6 Weeks Postpartum Among Obese Women (BMI $\geq$30 kg/m^2) by Race
eFigure 2. Fitted BP Trajectory (Solid Line) and 95% CI (Shaded Area) in First 6 Weeks Postpartum Among Women on No Antihypertensive Medications by Race
eFigure 3. Fitted BP Trajectory (Solid Line) and 95% CI (Shaded Area) in First 6 Weeks Postpartum Among Women on Antihypertensive Medications at the Time of Discharge by Race
eFigure 4. Fitted BP Trajectory (Solid Line) and 95% CI (Shaded Area) in First 6 Weeks Postpartum Among Women With Preeclampsia by Race

This supplementary material has been provided by the authors to give readers additional information about their work.

© 2020 Hauspurg A et al. *JAMA Network Open.*

eTable 1. Demographics of Overall Cohort

	**Overall Cohort N= 1,077**
Age (years)	30.1 (5.6)
Predelivery BMI (kg/m^2)	29.6 (7.9)
Gestational age at prenatal care establishment (weeks)	11.4 (6.3)
First prenatal systolic BP (mmHg)	118 (11)
First prenatal diastolic BP (mmHg)	74 (9)
Insurance status	
Private insurance	701 (65.1%)
Public insurance	357 (33.2%)
Other	19 (1.8%)
Primiparous	649 (60.3%)
Current tobacco use	117 (10.9%)
Pregestational diabetes	113 (10.5%)
Gestational diabetes	37 (3.4%)

Data are mean, SD unless otherwise specified

© 2020 Hauspurg A et al. *JAMA Network Open.*

eTable 2. Delivery and Postpartum Characteristics

	**Overall Cohort N=1,077**
Type of hypertension<br>Gestational hypertension<br>Preeclampsia	 447 (41.5%)<br>630 (58.5%)
Delivery by Cesarean section	473 (43.9%)
Birthweight (grams)	2930 (759)
Gestational age at delivery (weeks)	37.3 (2.8)
Highest systolic BP in 24 hours prior to discharge (mmHg)	139 (12)
Highest diastolic BP in 24 hours prior to discharge (mmHg)	88 (7)
Discharged on anti-hypertensives	262 (24.3%)
Seen for postpartum appointment	884 (82.1%)
Number of blood pressures reported through program, median [IQR]	17 [10-21]

Data are mean, SD unless otherwise specified

© 2020 Hauspurg A et al. *JAMA Network Open.*

eFigure 1. Fitted BP Trajectory (Solid Line) and 95% CI (Shaded Area) in First 6 Weeks Postpartum Among Obese Women (BMI ≥30 kg/m^2) by Race

Black women (shown in blue) and White women (shown in orange), (a) systolic and (b) diastolic

(a)

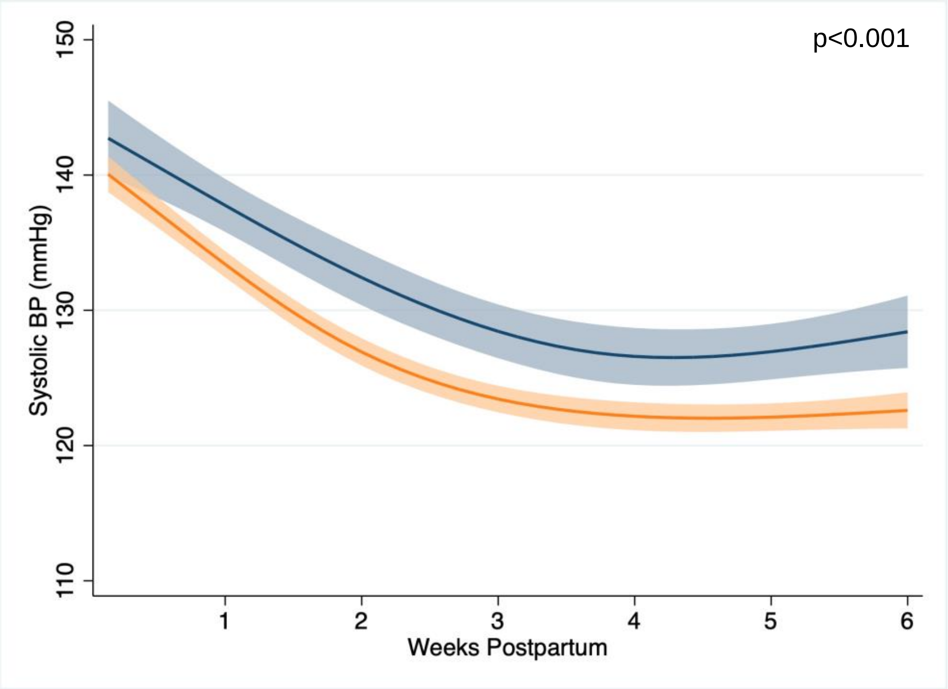

(b)

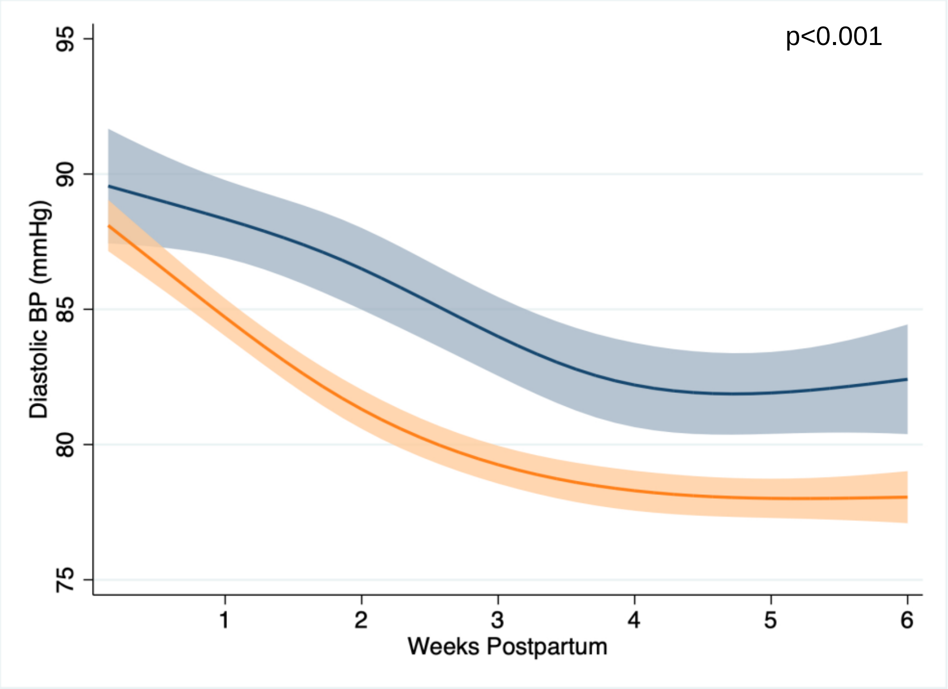

© 2020 Hauspurg A et al. *JAMA Network Open.*

eFigure 2. Fitted BP Trajectory (Solid Line) and 95% CI (Shaded Area) in First 6 Weeks Postpartum Among Women on no Antihypertensive Medications by Race

Black women (shown in blue) and White women (shown in orange), (a) systolic and (b) diastolic

(a)

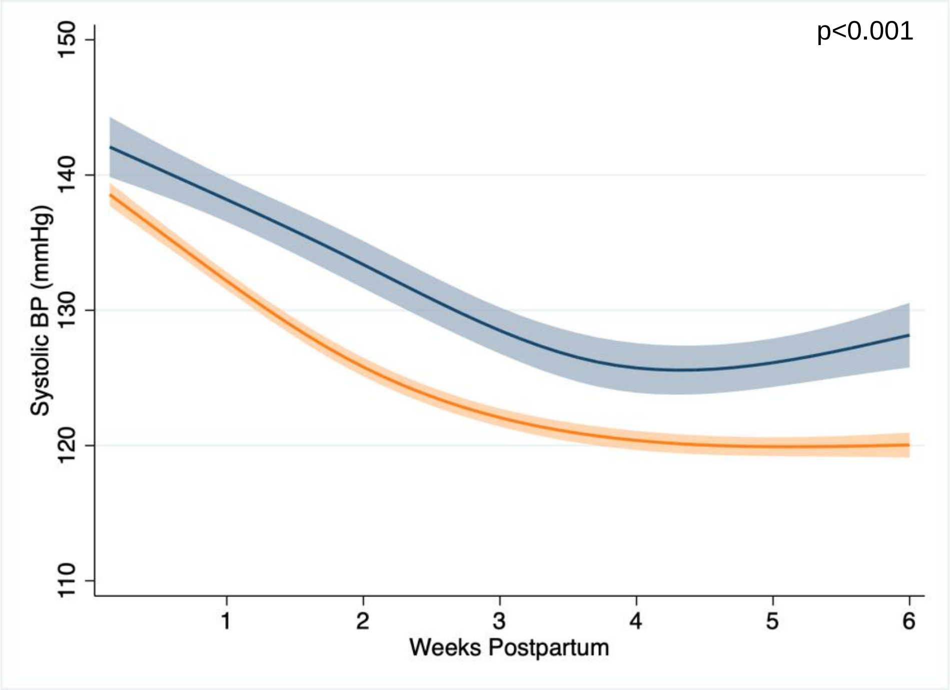

(b)

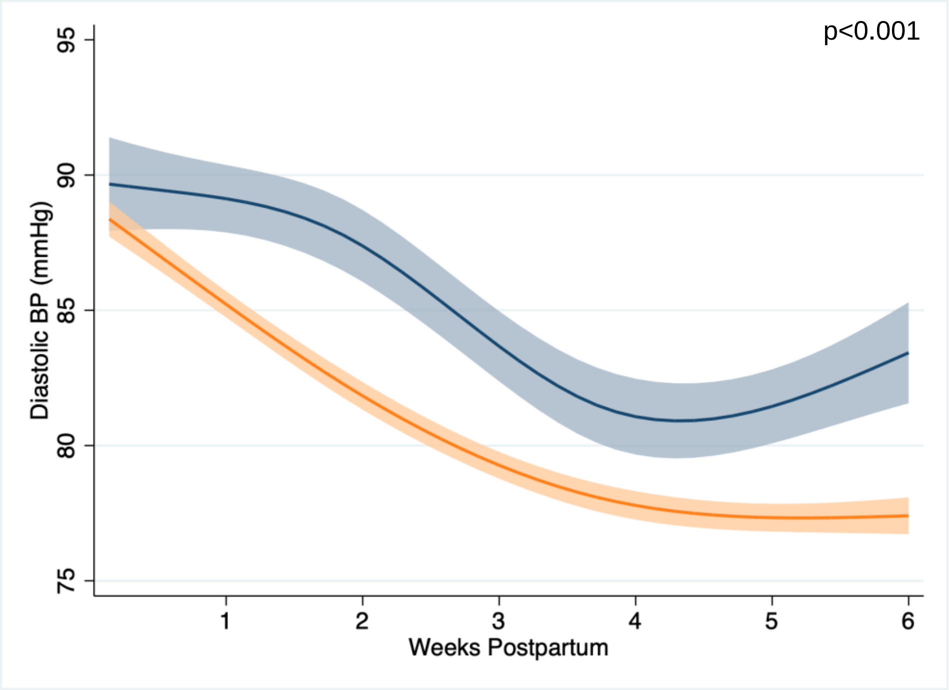

© 2020 Hauspurg A et al. *JAMA Network Open*.

eFigure 3. Fitted BP Trajectory (Solid Line) and 95% CI (Shaded Area) in First 6 Weeks Postpartum Among Women on Antihypertensive Medications at the Time of Discharge by Race; Black women (shown in blue) and White women (shown in orange), (a) systolic and (b) diastolic

(a)

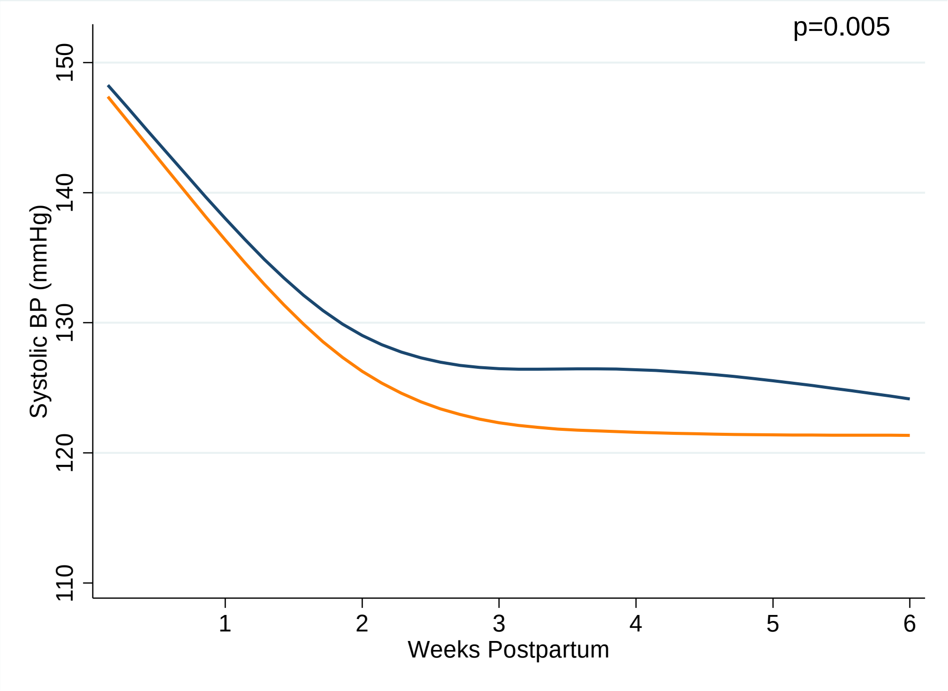

(b)

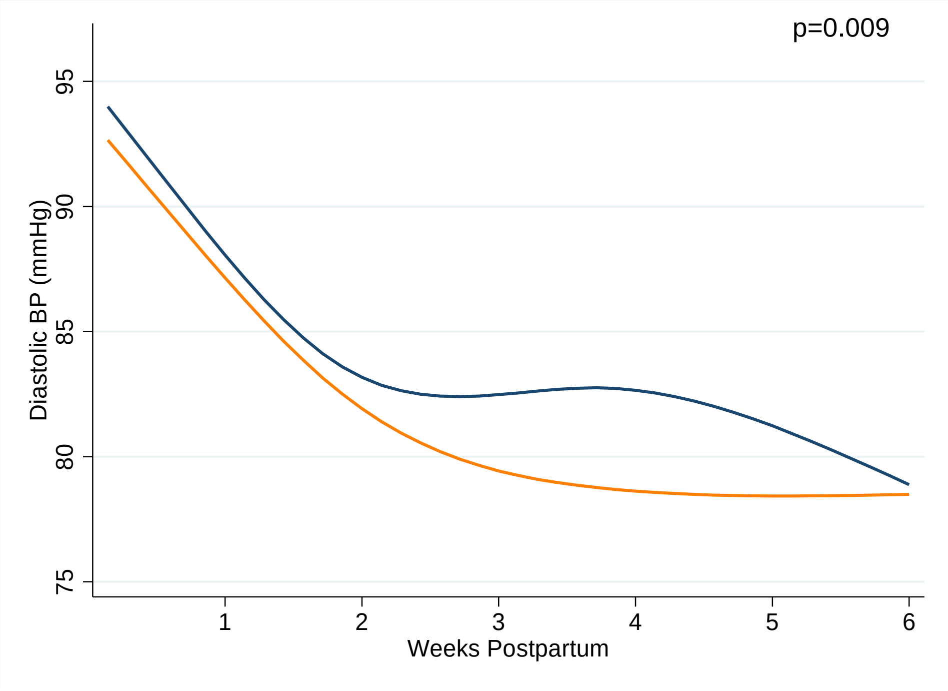

© 2020 Hauspurg A et al. *JAMA Network Open.*

eFigure 4. Fitted BP Trajectory (Solid Line) and 95% CI (Shaded Area) in First 6 Weeks Postpartum Among Women With Preeclampsia by Race

Black women (shown in blue) and White women (shown in orange), (a) systolic and (b) diastolic

(a)

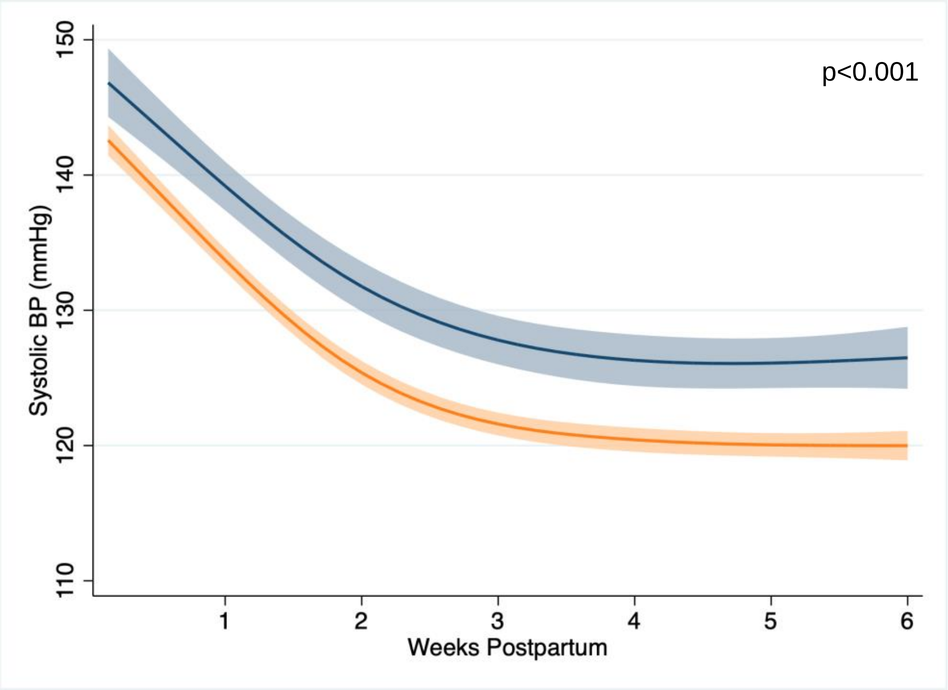

(b)

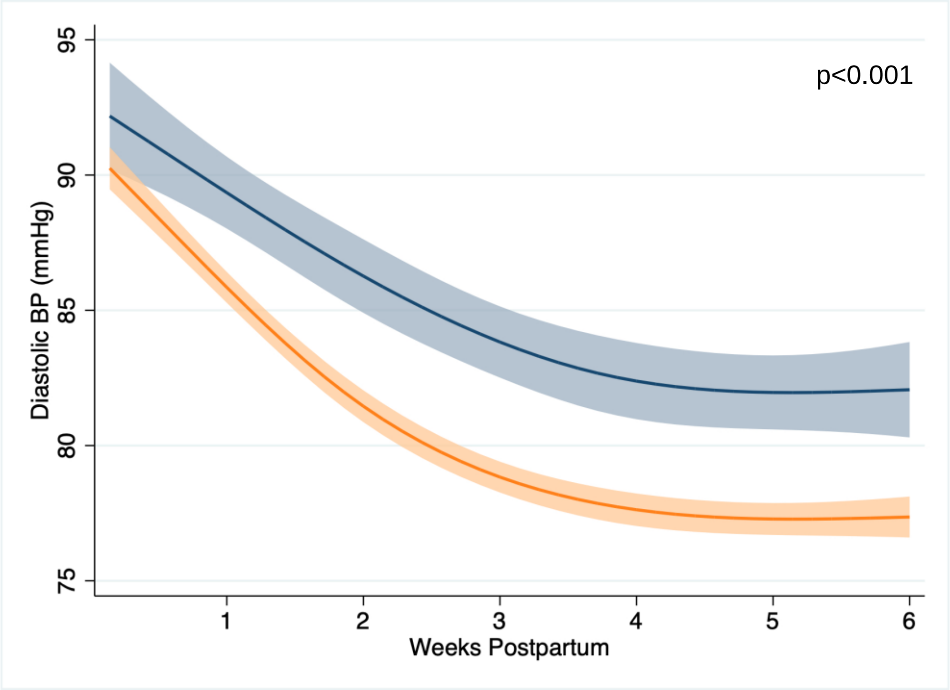

© 2020 Hauspurg A et al. *JAMA Network Open.*
